# Scaffold hunter: visual analysis of biological activity data

**DOI:** 10.1186/1758-2946-6-S1-P33

**Published:** 2014-03-11

**Authors:** Karsten Klein, Oliver Koch, Nils Kriege, Petra Mutzel, Till Schäfer

**Affiliations:** 1 School of Information Technologies, The University of Sydney, Australia; 2 Department of Chemistry and Chemical Biology, TU Dortmund, Germany; 3 Department of Computer Science, TU Dortmund, Germany

## 

**Figure 1:**
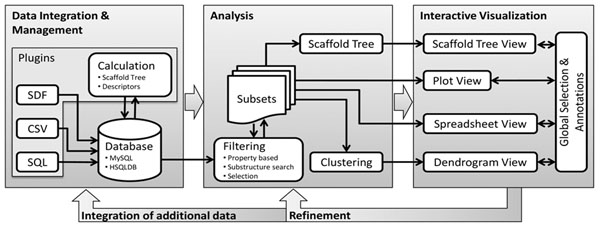
Iterative Workflow.

The growing interest in chemogenomics approaches over the last years has led to a vast amount of data regarding chemical and the corresponding biological activity space. The discovery of new chemical entities is not suitable to a fully automated analysis, but can greatly benefit from tools that allow exploring this chemical and biological space. We present a new version of Scaffold Hunter [1,2], a highly interactive tool that fosters the systematic visual exploration of compound and bioactivity data. The software supports the integration of data from various sources and provides several complementary analysis and visualization modules (Figure 1).

Scaffold Hunter features the scaffold tree algorithm to provide hierarchical classification schemes and offers several interconnected views reflecting different aspects of the data. As a further extension state of the art clustering techniques are now included that allow, for example, to create subsets based on fingerprint similarity. A key concept of Scaffold Hunter is to support a cyclic, iterative knowledge discovery process, where it is possible to refine subsets, adjust the parameters of analysis algorithms or the mapping of property values to visual attributes.

We give an overview over the various views, the workflow concept and present an exemplary analysis of screening datasets targeting *T. cruzi* and *T. brucei*, the causative agent of sleeping sickness and Chagas disease, respectively. Scaffold Hunter is platform-independent and freely available under the terms of the GNU GPL v3 at http://scaffoldhunter.sourceforge.net/.
